# Editorial: Targeting tumor EMT-related signaling by natural products

**DOI:** 10.3389/fphar.2023.1152328

**Published:** 2023-02-20

**Authors:** Sirajudheen Anwar, Nafees Ahmed, Letizia Giampietro

**Affiliations:** ^1^ Department of Pharmacology and Toxicology, College of Pharmacy, University of Hail, Hail, Saudi Arabia; ^2^ School of Pharmacy, Monash University Malaysia, Bandar Sunway, Selangor, Malaysia; ^3^ Department of Pharmacy, University G. d’Annunzio, Chieti, Italy

**Keywords:** natural products, epithelial-mesenchymal transition (EMT), targeting tumor, treatment of cancer, EMT inhibitors

Natural products represent a good resource for antitumor agents and an excellent starting point for the synthesis of natural compound derivatives, useful for the treatment of various types of cancer (Giampietro et al., 2021; Naeem et al., 2022). These include natural products from plant, marine, animal *etc.* (Anwar et al., 2022).

In the last few years, many natural products and their derivatives have been explored as cytotoxic, antiproliferative or antioxidant agents and have also shown antitumor effects in preclinical and clinical research (Boretti, 2022). In comparison to synthetic medications, natural compounds have a higher amount of stiffness, which enhances protein cross-talk. This leads to better fitting into the protein receptor, which helps in easy protein activation for efficient signalling. They are the ideal candidates against cancer due to their diversity and adaptable structure complexity, a special natural property for biological activities such as that of artemisinin and vincristine. (Atanasov et al., 2021). This unique property of natural compounds had led the researchers to improve the knowledge to explore how they affect the tumour microenvironment and tumour signalling pathways.

In particular, epithelial-mesenchymal transition (EMT) represents an actual goal for the study of the tumor progression. EMT is a biological process during which epithelial cells are converted in mesenchymal cells. In physiological conditions, EMT regulates different biological activity such as wound repair, embryogenesis and organ development (Huang et al., 2022). In atypical circumstances, EMT occurs during the normal physiological tissue repair process, leading to angiogenesis, fibrosis, loss of function, and carcinoma. EMT has been associated with the development of invasive and cancer stem cells in carcinomas. EMT is triggered by transcription factors, which comprise the core helix-loop-helix factors TWIST1 and TWIST2, as well as SNAIL (SNAI1) and SLUG (SNAI2). Natural products could provide unprecedented hope in the fight against EMT and expand therapeutic alternatives. E-cadherin and other epithelial-related proteins and genes might be upregulated as part of a natural compound’s EMT targeting strategy, whereas mesenchymal phenotypes like vimentin, fibronectin 1, and N-Cadherin could be downregulated (Anwar et al., 2022).

With this in mind, Liang et al. studied *in vivo* and *in vitro* effect of natural compound, methyl gallate (MG) in hepatocellular carcinoma (HCC). In this research, for the first time, it was demonstrated that MG inhibits the proliferation, migration, invasion, and the EMT of HCC cells by regulating matrix metalloproteinases (MMPs) and adenosine monophosphate-activated protein kinase AMPK/NF-κB pathway, particularly upregulated epithelial E-cadherin protein and downregulated mesenchymal vimentin protein. This study revealed that the natural compound MG may have potential to affect EMT and treat cancer.

An important study by Cao et al. demonstrates that total flavonoid aglycones extract (TFAE) isolated from *Scutellaria baicalensis* inhibits tumor growth and induces apoptosis. In particular, the researchers showed that baicalein, wogonin, and oroxylin-A were the active compounds of TFAE and obtained the reconstructed TFAE (reTFAE) that was able to inhibit the EMT process in A549 cells. In this study, the relationship between reTFAE and PI3K/Akt signaling pathways, with TWIST1 as the key protein, was elucidated by using bioinformatic technology. Moreover, stable isotope dimethyl-labelled proteomics technology was conducted to complement the follow-up mechanism that EMT-inhibition process may be realized through the glycolysis pathway demonstrating that TWIST1-targeted flavonoids could provide a new strategy to inhibit EMT progress for the treatment of non-small cell lung cancer (NSCLC).

Another use of natural compounds in anticancer therapy was studied by Kang et al. In this study, *Coptidis Rhizoma* 30% ethanol extract (CRE) was evaluated as antimetastatic agent showing a significant inhibition of HCT116/R cells migration and invasion. CRE showed a potential ability to inhibit cancer metastasis *via* the suppression of EMT in drug-resistant cells especially in Colorectal cancer (CRC). The mechanism of action consists of upregulation of the expression of an epithelial marker (E-cadherin) and downregulation of the expression of mesenchymal markers (vimentin, Snail, and ZEB2) at both the protein and gene levels. Moreover, anti-EMT activity of CRE and its related effects were associated with the CRE-mediated suppression of the TGF-β pathway, as shown by changes in the levels of downstream molecules (phosphorylated Akt and p38), and inhibition of migration, invasion, and protein expression of TGF-β after treatment/cotreatment with a TGF-β inhibitor (SB431542). In this research, berberine was found as a main component of CRE, and it was supposed that it might be a prime contributor to stop the EMT process. This study lays the foundations to consider *Coptidis Rhizoma* or berberine as therapeutic candidates for the treatment of metastasis and drug resistance in CRC.

Among different natural compounds studied for the prevention of various cancer, Gossypol showed antitumor effects against different carcinoma from 1984 (Tuszynski and Cossu, 1984) along with few adverse reactions such as hypokalemia, loss of appetite, nausea and other gastrointestinal effects. For these reasons, recently a gossypol derivative, apogossypolone (ApoG2), lacking two aldehyde groups, was studied as antitumor compound. In this context, Li et al. demonstrated that ApoG2 suppress significantly the cervical cancer (CC) cell proliferation, invasion and EMT process *in vitro* and *in vivo*.

Another natural compound possessing antioxidant activity and used in therapy and prevention of inflammatory diseases and cancer is caffeic acid (CA); it is present in many herbs, vegetables, and fruits. Alam et al. published an important review where the therapeutic potential of CA in cancer is reported. The authors reported that the anticancer activity of CA is mainly due to its prooxidant, and antioxidant properties connected with the prevention of reactive oxygen species formation, the decrease of the angiogenesis of cancer cells, the enhance of the tumor cells’ DNA oxidation, and the repression of MMP-2 and MMP-9. These effects were referred to many cancer types demonstrated by *in vivo* and *in vitro* tests. It was concluded that CA alone or in combination with other chemotherapeutic agents might be used to treat cancer.

In conclusion, this Research Topic highlights some of the more important and key aspects of the use of natural compounds and their derivatives in the treatment of cancer. In particular, in this Research Topic, targeting tumor EMT-related signalling pathway by natural products was reported pointing out the importance of this aspect by *in vivo* and *in vivo* studies. Original research and the review in this Research Topic help to understand the molecular mechanisms behind the antitumor effects of natural products useful to promote the development of promising therapeutic strategies. In the future, researchers will be able to identify natural compounds that act as EMT inhibitors, enhancing the chemosensitivity of cancer cells resistant to chemotherapy while preventing metastasis.
